# A pharmacological and brain imaging study of human vasopressin AVP1BR receptor functional polymorphisms

**DOI:** 10.1186/s12868-025-00963-7

**Published:** 2025-07-17

**Authors:** Adrián Alacreu-Crespo, Emilie Olié, Maxime Manière, Jeremy Deverdun, Emmanuelle Lebars, Maithé Corbani, Gilles Guillon, Philippe Courtet

**Affiliations:** 1 FondaMental Foundation, Créteil, France; 2 Department of Psychology and Sociology, Area of personality, Assessment and Psychological Treatment, University of Zaragoza, C/ Atarazana 4, Teruel, Aragón 44003 Spain; 3 PSNREC, Univ Montpellier, INSERM, CHU de Montpellier, Montpellier, France; 4 Department of Emergency Psychiatry and Acute Care, Lapeyronie Hospital, CHU Montpellier, Montpellier, France; 5 Institut de Génomique fonctionnelle, Univ Montpellier, CNRS, INSERM, Montpellier, France; 6 Department of Neuroradiology, Academic Hospital of Montpellier & U1051, Institut of Neurosciences of Montpellier, Montpellier, France; 7 I2FH, Institut d’Imagerie Fonctionnelle Humaine, Montpellier University Hospital, Gui de Chauliac Hospital, Montpellier, France

**Keywords:** Vasopressin, Vasopressin 1B receptor, Genetic polymorphism, Pharmacological signaling, Emotional processing, fMRI

## Abstract

In humans, vasopressin AVP1BR receptor (hV_1B_) plays key roles in hypothalamic–pituitary–adrenal (HPA) axis regulation and social behavior. Three hV_1B_ polymorphisms, rs35369693 (K65N), rs28632197 (R364H) and rs33990840 (G191R), have been related to psychiatric disorders with altered HPA axis function and social behavior. The aim of this study was to explore hV_1B_ pharmacological properties as a function of the polymorphism in transfected cells and the brain functioning in an emotional task in volunteers harboring different AVP1BR polymorphisms. Transfection rate, fluorescent imaging and inositol phosphate (IPs) accumulation were evaluated in HEK293 cells that expressed different hV_1B_ variants: K65/G191/R364 (wild type), G191R, K65N and/or R364H. Brain functional activity was investigated in 35 healthy men with different hV_1B_ variants during an fMRI implicit emotional recognition paradigm. IPs accumulation after arginine vasopressin stimulation was much reduced in cells expressing hV_1B_ K65N and/or R364H, and increased in cells expressing G191R. Basal IPs accumulation, transfection rate, and fluorescent binding to plasma membrane were similar for all polymorphisms. During the anger vs. neutral face visualization task, activation of motor areas, visual areas, frontal sub-gyral area, hippocampus, and putamen was higher in homozygotes for the K65/R364 haplotype than in heterozygotes. Analyses did not include participants with the G191 polymorphism because of its low frequency. Different hV_1B_ polymorphisms could be candidates as biomarkers of psychiatric disorders. Moreover, hV_1B_ may be a pharmacological target if these polymorphisms are considered.

## Introduction

Arginine vasopressin (AVP) displays physiological antidiuretic and pressor effects [1]. AVP also has key roles in the regulation of complex social cognition and behaviors in different mammals [2], and is increasingly recognized as an important modulator of the hypothalamic–pituitary–adrenal (HPA) axis [3]. HPA axis activation by exposure to stress first elicits the release of corticotrophin-releasing hormone (CRH) and AVP, and then triggers the release of glucocorticoids in the adrenal cortex. AVP acts synergistically with CRH to mediate the stress response initiated in the anterior pituitary gland and modulates adrenocorticotrophic hormone (ACTH) secretion [4].

AVP effects are mediated through G-protein-coupled receptors that can be divided into three subtypes: AVP1AR, AVP1BR, and AVP2R (hV_1A_, hV_1B_, and hV_2_, respectively, in humans) [5]. The hV_1B_ receptor [6] was discovered in the rat hypophysis. It is directly coupled to phospholipase C (PLC) which leads, upon AVP stimulation, to both diacylglycerol and inositol tris-phosphate (IP_3_) accumulation. Both these second messengers trigger the biological effects associated with AVP *via* the activation of Proteine Kinase C and to a rapid increase of intracellular calcium concentration [7, 8]. The hV_1B_ receptor is important in HPA regulation, because it mediates AVP induction of ACTH secretion [6] and AVP-mediated CRH action potentiation [9].

Importantly, hV_1B_ may be involved in several neuropsychiatric disorders through its role in HPA dysregulation and social behavior [10]. In humans, some studies have associated single nucleotide polymorphisms (SNPs) and haplotypes in hV_1B_ with several personality traits and neuropsychiatric disorders. Specifically, a SNP (rs35369693) leading to an amino acid change at position 65 of hV_1B_ (K65N) has been related to behavioral and mood disorders in childhood [11, 12]. Another SNP (rs28632197) leading to an amino acid change at position 364 (R364H) has been associated with adult panic disorder [13] and aggression problems in children [11]. These two SNPs rarely co-exist. Moreover, a third SNP (rs33990840) leading to an amino acid change at position 191 (G191R) has been associated with suicide attempts in individuals with severe depression [14]. A better understanding of the impact of the different hV_1B_ variants (i.e. genotype-phenotype relationships) may help to develop specific pharmacological interventions for suicidal behaviours.

In addition, neuroimaging studies may shed light on the neural mechanism by which AVP regulates social stress responses [15–17] and behavioural responses to social stimuli [18]. Previous studies using functional magnetic resonance imaging (fMRI) have shown that AVP modulates the limbic system response to facial emotional stimuli [19]. Moreover, AVP increases amygdala functional connectivity with the anterior insula [20], which may increase the ability of amygdala to induce visceral somatic markers that guide decision-making.

The translational interest of V_1B_R and its coding gene hV_1B_ may be supported by studies to characterize in vitro the pharmacological properties of the different hV_1B_ variants and particularly by imaging studies in humans to relate common variants to brain function. The obtained data on the effects of the different genetic variants might help to understand the link between genotype and behavior. To our knowledge, no pharmacological data are currently available on hV_1B_ polymorphisms. In the present study, we explored three hV_1B_ polymorphisms (rs35369693, rs28632197, rs33990840) to determine their possible contribution to stress-related disorders using two complementary approaches. First, to study the pharmacological properties of the different hV_1B_ variants, we explored their couplings to phospholipase C by measuring the total Inositol phosphates which accumulated under AVP stimulation, which reflect the initial Inositol tri phosphate production [8]. Second, we carried out an explorative fMRI study in healthy controls to determine whether hV_1B_ genetic variants modulate brain activation by emotional stimuli.

## Methods

### Pharmacological study

#### Generation of cDNA constructs

Plasmids carrying wild type (WT) and hV_1B_ variants were constructed and expressed in HEK293 cells (obtained from the American Type Culture Collection (ATCC)) as previously described [21]. Briefly, DNA constructs were generated in the pDNA3.1 expression vector by inverted PCR amplification (Phusion High Fidelity Polymerase, Fisher Bioblock Scientific, Illkirch, France) using specific primers including the single amino acid variants and full-length wild type (WT) human hV_1B_ cDNA as template (kindly provided by Maria-Angeles Ventura, Institut Cochin, Paris, France) [22]. The primers used were: 5’-CTG GGC CGC AAC CGC TCC CGC-3’ and 5’-CTG GCC CAG GGT CAG CAG CAC-3’ to obtain hV_1B_ harboring the K65N variant; 5’-GGC AGA CTT CCG CTT CC-3’ and 5’-CAG CAG TCC AGC ACC CC-3’ to obtain V_1B_R harboring the G191R variant; and 5’-GCC CAG GAT GCA CCG GC-3’ and 5’-TGG GGA CCC CCA CAG CAG GCA-3’ to obtain hV_1B_ harboring the R364H variant. To generate the double mutant K65N/R364H, the cDNA of the K65N mutant was used as template to add the second mutation using the R364H primers. After inverted PCR amplification, DNA was digested with *DpnI* before agarose gel electrophoresis. Then, only the band corresponding to the full length DNA for the variant was collected (Geneclean, Bio101, MenloPark, USA) and ligated (T4 ligase NEBiolabs, Evry, France). After verification by restriction enzyme mapping, all constructs were sequenced to verify the proper directional in-frame insertion and sequence integrity (Beckman Coulter Genomics, Essex, UK).

#### IP accumulation

The total inositol phosphate amount which accumulated under hormonal stimulation (IPs) was determined as described previously [21]. As further demonstrated, IP_S_ we measured in our assays reflects the initial production of Inositol triphosphate the well-known second messenger generated upon AVP1AR and AVP1BR Vasopressin receptors activation (Kirk et al., 1986). HEK293 cells were transiently transfected with WT or hV_1B_ variants by electroporation. Then, were plated in DMEM (Invitrogen, Cergy Pontoise, France) with 10% fetal calf serum (maker) and penicillin-streptomycin (Invitrogen, Cergy Pontoise, France) for 24 h. Then, cells were incubated in inositol-free DMEM medium (ICN Biochemicals, Orsay, France) with 1 µCi/ml myo-[2-^3^ H] inositol (20 Ci/mmol; from Perkin Elmer, Courtaboeuf, France) for 24 h. On experiment day, cells were stimulated with increasing concentrations of AVP (Bachem, Bubendorf, Switzerland) for 15 min. Reaction was stopped by addition of perchloric acid (5% vol/vol). Total IP_S_ were extracted, purified by exchange chromatography using Dowex AG1-X8 formate form 200–400 mesh (Bio-Rad, Munich, Germany), and quantified. Free myo-[2-^3^ H] inositol also was measured in each condition to normalize the results to cell number/well. In some experiments, total radiolabeled inositol lipids where also measured for the determination of basal PLC activity [8].

#### Cell labeling and microscopy

Transfected HEK293 cells were plated on 12 mm glass coverslips pre-coated with poly-ornithine and grown for 2 days. Selective hV_1B_ receptor labelling was performed using a selective hV_1B_ receptor agonist d[Leu^4^-(Lys-Al647)^8^]VP previously pharmacologically characterized [23]. Transfected cells were incubated with 150 nM of d[Leu^4^-(Lys-Al647)^8^]VP in a DMEM medium containing: 0.2 mg/mL BSA, 25 mM HEPES, pH 7.4, at 12 °C for 1 h [23]. After three washes in cold PBS, cells were fixed in 4% paraformaldehyde at 4°C overnight. Coverslips were mounted with Mowiol. Fluorescent cells were imaged using a confocal microscope Zeiss LSM510 Meta and the helium/neon laser 633 nm for excitation of Alexa 647, and the LP650 nm filter for emission. Images were acquired using a 40x (NA 1.4) objective for oil immersion at zoom 0.7x for counting transfected cells and at zoom 3x for detailed observation. Membrane fluorescence was evaluated on 30–40 cells for each experimental conditions by the Zeiss LSM Browser and Image J using the 1-250 fluorescent unit dynamics. Results are the mean of at least 3 distinct experiments in which 30–40 cells were evaluated for each experimental conditions as previously described [21, 24]. To determine the level of receptor transfection, we also generated contrast phase images of each fluorescent cells. The receptor, transfection rate was calculated as the % the cells exhibiting a specific fluorescent labelling (fluorescence imaging) compared to the labelled plus unlabeled cells presents in the same coverslip area (contrast phase imaging). The fluorescence associated to plasma membrane for hV_1B_ receptors mutants was determined by measuring the intensity of Alexa 647 fluorescence present on a similar plasma cell membrane area using the same fluorescent microscope setting and expressed as Arbitrary Units (AU) [23].

#### Statistical analysis

IPs accumulation data were analyzed using the GraphPad (GraphPad Software, Inc., San Diego, CA). K_act_, the concentration of AVP leading to half-maximal PLC stimulation and E_max_, the maximal hormonal stimulation rate was calculated using GraphPad Prism. Results are expressed as the mean ± SEM of at least 3 distinct experiments each performed in triplicate. Statistical analysis of the data was performed using the one-way ANOVA test using hV_1B_ polymorphism as independent variable. Homogeneity of variance was assessed with the Bartlett test. Pairwise post-hoc comparisons between the wild type and the other variants were assessed using Tukey correction.

### fMRI study

#### Participants

The 35 healthy men were from a previous study conducted by Olié et al. [25]. Participants were recruited from a list of volunteers from the Montpellier Academic hospital via the Centre of Clinical Investigation, Montpellier University Hospital. They had no history of psychiatry disorders according to the Diagnostic and Statistical Manual of Mental Disorders, Fourth Edition (DSM-IV), criteria. An experienced psychiatrist (EO) confirmed that they met the inclusion criteria: free of a lifetime history of mental health disorder. Exclusion criteria included neurological disorders, head injury, consumption of psychotropic drugs. All participants were euthymic at fMRI time, as indicated by the Hamilton Depression Rating Scale [26] score < 7. The French version of the National Adult Reading Test [27] was used to assess their verbal intelligence quotient, the Spielberger State and Trait Anxiety Scale (STAI) [28] to determine their current level of anxiety, and the State-Trait Anger Expression Inventory (STAXI) [29] to evaluate their current level of anger and trait anger.

The local Ethics Committee (CPP Sud Mediterranée IV, CHU Montpellier) approved the study protocol according with the declaration of Helsinki. All participants provided a written informed consent before enrollment.

#### fMRI facial emotion paradigm

Each participant completed three consecutive 6-minute scanning runs including photographs from standardized series of prototypical faces expressing happiness, anger, or sadness from ten different individuals. Each run included 20 photographs of faces expressing one of these three emotions and 20 neutral faces in a randomized order. The order of runs was counterbalanced across participants and groups. The interstimulus interval varied from 3 to 8 s according to a Poisson distribution to prevent participants from predicting the timing of the next stimulus. During the interstimulus interval, participants viewed a fixation cross. Participants viewed each face for 2 s and were asked to identify the face sex. The reaction time corresponds to the interval between the stimulus start and time of sex identification (by pressing the relevant button). No reference to emotion was made during the instructions.

#### fMRI acquisition

Imaging acquisition was done using a 1.5T whole-body MRI system (MAGNETON AVANTO, Siemens, Erlangen, Germany) equipped with a standard 12-channel receive-only head coil. During the facial emotion paradigm, 180 volumes of BOLD gradient-echo, echo planar images (GE-EPI) were obtained. GE-EPI characteristics were TR = 2 s, TE = 40 ms FOV = 220 mm, 25 axial slices (5 mm slice thickness), slice gap = 0.5 mm, voxel size = 3.43 × 3.43 × 5 mm3, and flip angle 90°. Slices covered a region extending from the vertex to the cerebellum lower parts. A 3DT1 gradient-echo sequence (3DT1 Flash, TR/TE 11/5.2ms, 15° flip angle), aligned with corpus callosum, was obtained for each participant (voxel-size 0.93 × 0.938 × 1 mm, 160 transversal slices).

#### Blood samples

Venous blood samples were collected at inclusion. Genomic DNA was extracted from peripheral blood leukocytes with the Nucleon BACC2 Genomic DNA Extraction Kit (GE Healthcare, Glattbrugg, Switzerland). To detect the SNPs rs35369693 (K65N) in exon 1, rs33990840 (G191R) in exon 1, and rs28632197 (R364H) in exon 2 of hV_1B_, flanking exon primers from published sequences were designed with the Primer3 (ver. 0.4.0) online program (http://frodo.wi.mit.edu/cgi-bin/primer3/primer3_www.cgi). PCR conditions are available on request. PCR products were purified with the QIAquick PCR Purification Kit (Qiagen, Germantown, MD, USA) and sequenced on an ABI PRISM 3100 Genetic Analyzer (PE Applied Biosystems, Foster City, CA, USA).

#### Statistical analyses

fMRI data were analyzed using SPM12 (Wellcome Department of Imaging Neuroscience, London, UK) implemented in Matlab 2015 (Mathworks, Inc., Natick, MA) using an event-related model. The first volumes of each fMRI run were discarded due to unsteady MRI signal. EPI data were first corrected for head movements, spatially normalized into a standard stereotactic space (Montreal Neurological Institute T1 template), and smoothed using an isotropic 8 mm full-width-half-maximum Gaussian kernel. Images were reconstructed with a voxel size = 3 × 3 × 3 mm^3^. Each normalized image set was also band-pass filtered with a 128s temporal high pass filter to remove low-frequency noise. Then, brain area activation elicited by emotional vs. neutral faces in each run (angry, sad, and happy) were analyzed to identify differential activations between genotype groups (GG homozygotes vs. heterozygotes) for each polymorphism. The threshold for the median activation values was set at an uncorrected *p* <.001 with a minimum cluster size of 25 voxels.

Other statistical analyses (not concerning the fMRI data) were carried out with SPSS 22.0 (SPSS, Inc., Chicago). As the groups were small in size, only non-parametric tests were used. Quantitative data were compared between groups with the Mann-Whitney U test. Differences were considered significant at *p* <.05.

## Results

### Pharmacological characterization of the hV_1B_ variants rs35369693 (K65N), rs28632197 (R364H) and rs33990840 (G191R) variants

**Fig. 1:**
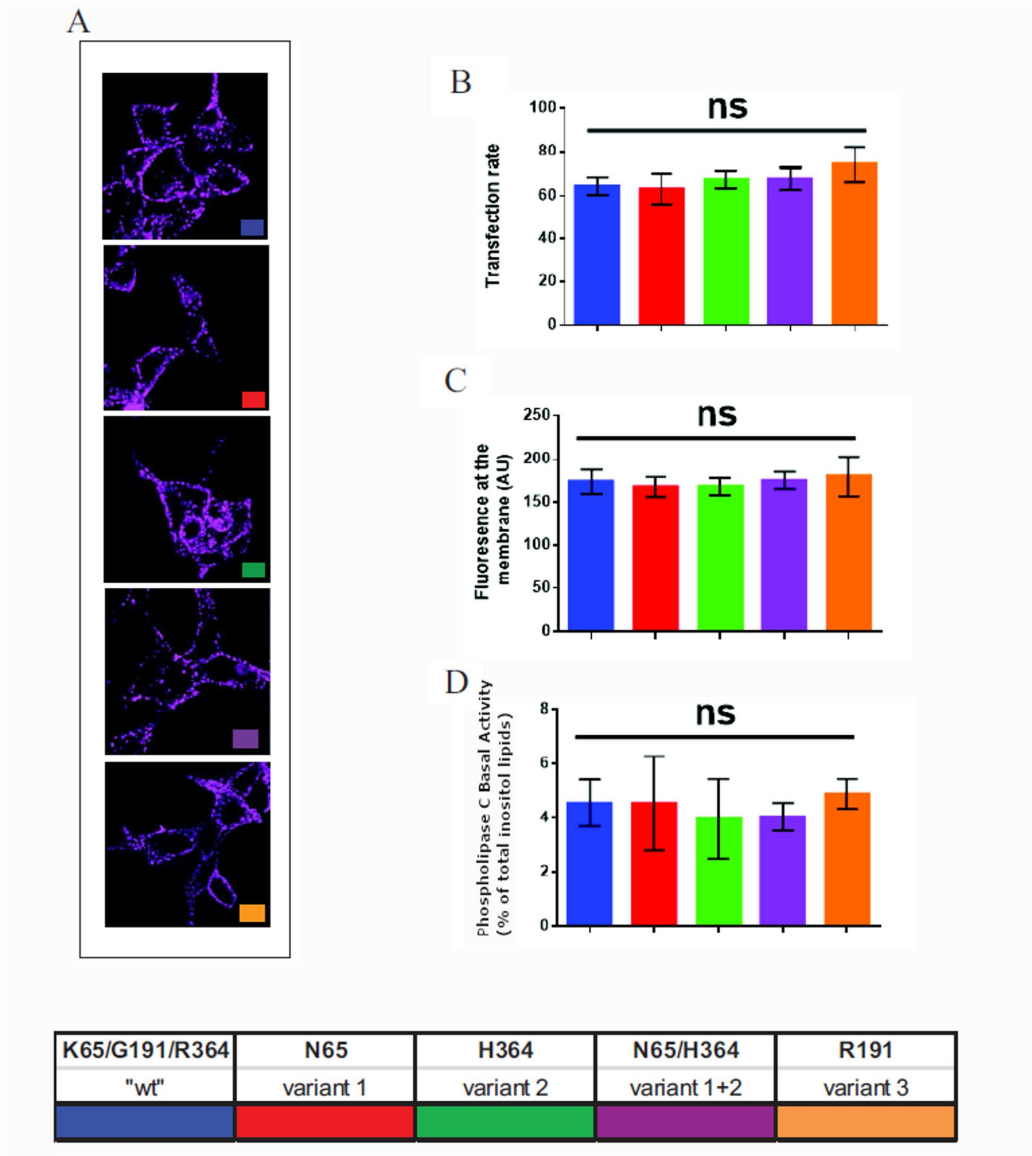
Expression of the different hV_1B_ receptor variants expressed in HEK293 transfected cells. HEK293 cells were transfected with the different hV_1B_ receptor variants and, as control, with the wild type hV_1B_ receptor (K65/G191/R364). Transfected cells were incubated with a saturating concentration of the selective fluorescent hV_1B_ analogue d[Leu^4^-(Lys-Al647)^8^]VP as described under Methods (panels **A**, **B**, **C**) and used for fluorescence imaging experiments. Data are representative of at least 3 distinct transfection experiments for which more than 100 fluorescent individual transfected cells were analyzed (panels **A**, **B**, **C**). Statistic: ns: p-value > 0.05. In panel D transfected cells were pre -incubated with tritiated inositol to measure PLC activity. Panel **A**: Illustration the specific labelling of each transfected hV_1B_ receptors in HEK293 cells using dLeu^4^-(Lys)^8^]VP as specific ligand (see methods). Non-specific binding controls were performed under the same experimental conditions. In the presence of 1 micro molar of AVP, no significant fluorescence labelling was observed (data not shown). Panel **B**: The transfection rate of the different hV_1B_ receptors in HEK293 cells prelabelled with d[Leu^4^-(Lys-Al647)^8^]VP was determined as described under Methods. Panel **C**: The expression of the different hV_1B_ receptors to HEK293 plasma membrane were determined as described under Methods. Results are expressed as Arbitrary Unit. Panel D: The level of basal Phospholipase C activity of transfected hV_1B_ receptors was measured. Results were expressed as the % of total labelled Inositol Lipids converted into labelled Inositol Phosphate during a 30 min incubation period at 37 °C as described under Methods

First we controlled that, upon receptors transfection, the different hV_1B_ were correctly addressed to the HEK293 plasma membrane. Transfected cells were incubated with a selective hV_1B_ fluorescent ligand and subjected to fluorescent imaging. As illustrated in Fig. 1 panel A, regardless of which hV1B was transfected, the specific labelling was only observed at the cell periphery corresponding to plasma membrane. Moreover, this labelling was specific since it completely disappeared in the presence of 100nM AVP (data not shown). The rate of cell transfection was then calculated as described under methods and illustrated Fig. 1 panel B. This rate (around 70%) was found statistically identical whatever the hV_1B_ used for transfection (F_4,10_ = 0.79, *p* =.553). Similarly, the density of transfected hV_1B_ associated to plasma membrane was quantified by fluorescent imaging. It was found statistically identical (F_4,10_ = 0.15, *p* =.959) for all the hV_1B_ tested (Fig. 1, panel C). Finally, we measured the basal PLC activity of each batch of hV_1B_ receptors transfected cells. These values were found statistically similar (F_4,10_ = 0.127, *p* =.969) whatever the transfected hV_1B_ considered (Fig. 1, panel D) indicating that, under our experimental conditions, the number of transfected cells par assay was statistically similar. Altogether these controls indicate that, upon receptors transfection, the different hV_1B_ were properly addressed to the HEK293 plasma membrane and expressed in similar densities. We thus could compare the potential differences of hV_1B_ coupling using this model of transfected cells.

**Fig. 2:**
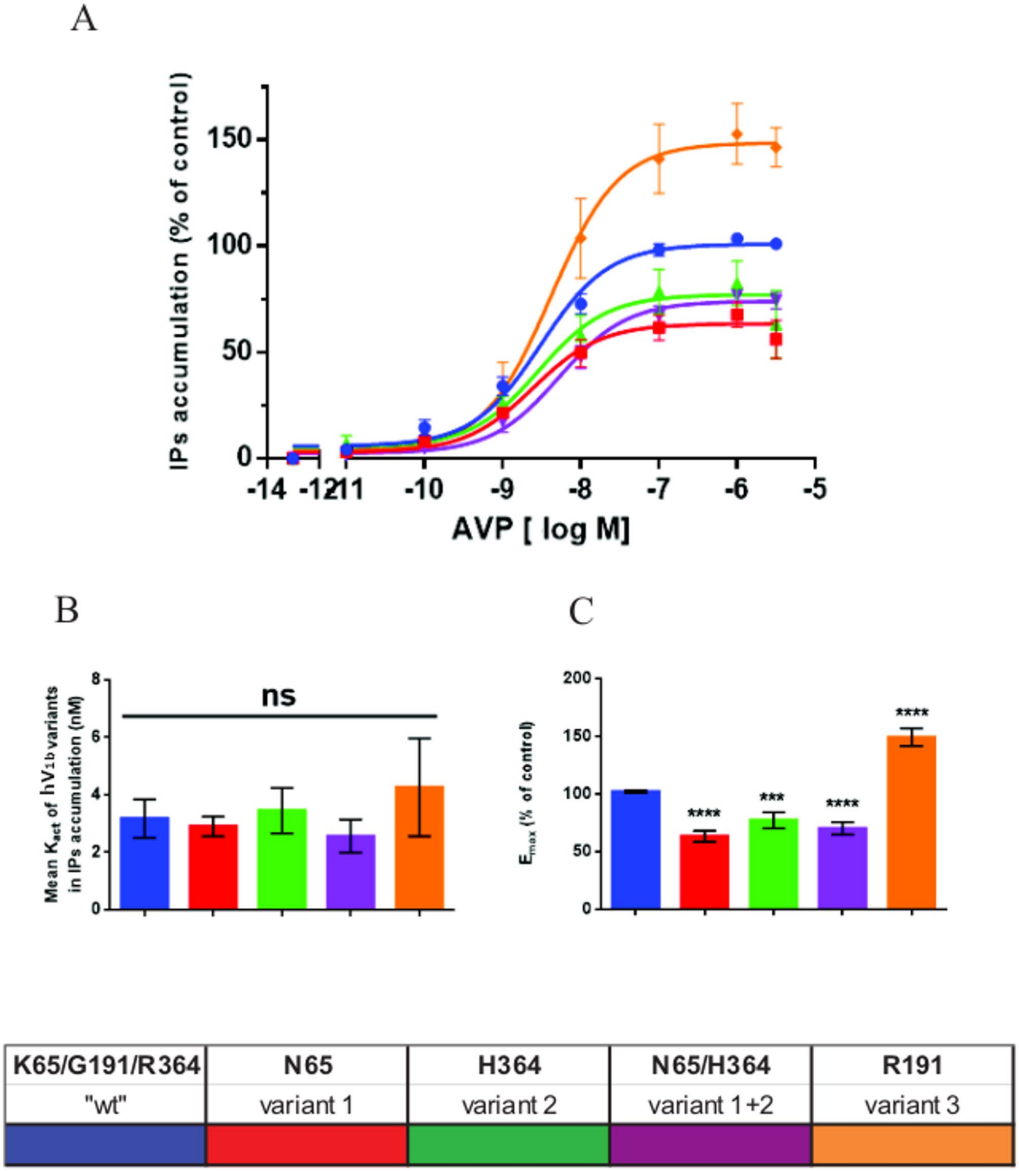
Pharmacological properties of the different hV 1B receptors expressed in HEK2393 transfected cells: Phospholipase C assays. Panel **A** HEK293 cells were transfected with each hV_1B_ receptors variants and with the wild type hV_1B_ receptor (K65/G191/R364) as control. Transfected-cells were first grown 24 h in the presence of labelled Inositol to measure their PLC activity and further incubated 30 min at 37 °C with or without increasing amounts of AVP in the presence of Chloride Lithium to prevent Inositol phosphates dephosphorylation. Total IPs which accumulated during incubation with or without AVP (basal condition) was measured and expressed as % of maximal PLC activity measured with control transfected cells under the same experimental conditions. Panel **B** illustrated the concentration of AVP leading to half maximal IP_S_ accumulation (K_act.,_ expressed in nM) for both each hV_1B_ receptors variants and control cells. Results are deduced from experiments illustrated in panel **A**. 100%= ù. Panel **C** illustrated the maximal IPs accumulation (Emax) induced by AVP and expressed as % of Emax from control transfected cells for both each hV1B receptors variants. Results are deduced from experiments illustrated in panel A Statistics: Each experiment was performed in triplicate and data illustrated are the mean + _ SEM of at least 3 independent experiments. ns = p-value > 0.05; *=0.01 < p-value < 0.05; **= 0,001 < p-value < 0.01; ***=0.0001 < p-value < 0.001; ****= p-value < 0.0001

hV_1B_ coupling to PLC was then evaluated by quantifying IPs accumulation in HEK293 transfected cells expressing the WT hV_1B_ (K65/G191/H364) and each non-synonymous variant upon stimulation with increasing doses of AVP. As illustrated in Fig. 2, panel A, whatever the transfected hV_1B_ used, AVP dose dependently stimulated IPs accumulation with a saturation observed for around 100nM AVP. ANOVA for the concentration of AVP leading to half maximal stimulation (Kact) showed significant differences between groups (F_4,10_ = 3.59, *p* =.046). However, as illustrated in Fig. 2, panel B, post-hoc comparisons showed not significant differences between the wild type compared with other variants of the hV_1B_ tested (3.0 ± 0.3 nM for K65/G191/H364, 2.4 ± 0.5 nM for K65N, 2.9 ± 0.9 nM for R364H, 5.2 ± 0.6 nM for K65N/R364H, and 4.1 ± 0.5 nM for G191R; all *p* >.050). By contrast maximal, IPs accumulation (E_max_) observed upon AVP stimulation was significantly decreased (F_4,10_ = 37.78, *p* <.001) in cells expressing the K65N, R364H, or K65N/R364H variant (by 34.7%, *p* <.001 for K65N; by 22.7%, *p* <.003 for R364H; by 29.9%, *p* <.001 for K65N/R364H respectively) compared with cells expressing the WT receptor (Fig. 1, panel C). Conversely, maximal IPs accumulation was increased in cells expressing the G191R variant (increase by 49%, *p* <.001) compared with cells expressing WTV_1B_R.

### Effect of hV_1B_ polymorphisms on fMRI emotional processing

#### Description of sample

As the hV_1B_ genotype was not available for one participant, data from only 34 volunteers were analysed. Their median age was 39 years (min-max: 31–49) and their mean number of years of education was 15 (min-max: 11–18).

Twenty-eight participants (82.4%) carried homozygote alleles (GG) for hV_1B_ rs35369693 (K65N) and six carried heterozygote alleles (GC). Twenty-seven participants (79.4%) carried homozygote alleles (GG) for hV_1B_ rs28632197 (R364H) and seven carried heterozygote alleles (AG). Thirty-one participants (91.2%) carried homozygote (GG) and three harboured heterozygote (GC) alleles for hV_1B_ rs33990840 (G191R). Therefore, for the next analyses, only the genotype for hV_1B_ rs35369693 (K65N) and rs28632197 (R364H) were considered.

Sociodemographic variables and psychological measures were not different between genotypes for the hV_1B_ rs35369693 (K65N) and rs28632197 (R364H) SNPs (Table 1).

**Table 1:** Comparison of sociodemographic data, clinical variables and reaction times (mean ± SEM):

	hV_1B_ rs35369693 (K65N)			hV_1B_ rs28632197 (R364H)		
	GG	GC	*p value*	GG	AG/AA	*p value*
N=	28	6		27	7	
Age	40.86 ± 1.06	37.50 ± 2.01	0.100	40.96 ± 1.09	37.57 ± 1.70	0.081
Years of study	15.00 ± 0.38	14.67 ± 0.84	0.809	15.07 ± 0.39	14.43 ± 0.75	0.478
NART	25.43 ± 1.06	22.17 ± 2.59	0.220	25.33 ± 0.68	23.00 ± 2.34	0.478
Hamilton score	0.39 ± 0.13	0.33 ± 0.33	0.741	0.41 ± 0.13	0.29 ± 0.29	0.617
STAI trait	40.44 ± 2.89	46.17 ± 3.09	0.174	41.00 ± 2.96	43.42 ± 3.79	0.444
STAI state	32.46 ± 2.77	36.00 ± 3.28	0.264	32.80 ± 2.87	33.43 ± 3.19	0.394
STAXI trait	16.72 ± 1.18	17.83 ± 2.61	0.644	16.78 ± 1.23	17.43 ± 2.25	0.617
STAXI state	11.85 ± 0.69	12.40 ± 0.98	0.214	11.92 ± 0.72	12.00 ± 0.89	0.478
RT Angry faces	971 ± 32	761 ± 125	0.131	975 ± 33	778 ± 107	0.083
RT Happy faces	898 ± 29	721 ± 115	0.188	900 ± 31	739 ± 99	0.163
RT Sad faces	813 ± 59	769 ± 142	0.581	816 ± 62	763 ± 119	0.379
RT Neutral faces	882 ± 44	709 ± 116	0.159	882 ± 46	733 ± 101	0.177

*NART* National Adult Reading Task, *STAI * State-Trait Anxiety Scale, *STAXI* State-Trait Anger Expression Inventory, *RT*  Reaction Time

#### fMRI analyses

The median reaction times for emotional faces and neutral faces were not different in function of the hV_1B_ genotypes (*p* >.050).

In whole brain fMRI analyses, activation of the right supplementary motor area (*p* <.001, k = 19), left hippocampus (*p* <.001, *k* = 16), right putamen (*p* <.001, *k* = 32), right temporal middle gyrus (*p* <.001, *k* = 19), left frontal sub-gyral (*p* <.001, *k* = 16), right temporal inferior (*p* <.001, *k* = 25), and right precuneus (*p* <.001, *k* = 18) areas was increased in people with the GG genotype compared with the CG genotype for hV_1B_ rs35369693 (K65N) when viewing angry vs. neutral faces (Table 2). Moreover, activation of the supplementary motor area (*p* <.001, *k* = 15) and left lingual gyrus (*p* <.001, *k* = 38) was increased in people with the GG genotype compared with participants with the AG/AA genotype for hV_1B_ rs28632197 (R364H) during the task visualization of angry vs. neutral faces.

**Table 2:** Comparison of brain activation during the fMRI angry vs. neutral emotion paradigm, voxel *p* <.001, k ≥ 15:

	K_E_	T	MNI (x, y, z)	Area
hV_1B_ rs35369693 (K65N) GG > CG	19	5.09	(12, -1, 67)	Sup motor area (R)
	16	4.90	(-30, -31, -11)	Hippocampus (L)
	32	4.64	(33, − 10, 1)	Putamen (R)
	19	4.35	(42, -73, 19)	Temporal mid (R)
	16	4.27	(-21, -31, 46)	Frontal sub-gyral (L)
	25	4.21	(54, − 52, -5)	Temporal inf (R)
	18	4.19	(9, -61, 52)	Precuneus (R)
hV_1B_R rs28632197 (R364H) GG > AG/AA	15	5.18	(12, − 4, 64)	Sup motor area (R)
	38	4.63	(-15, − 85, -14)	Lingual gyrus (L)

*Sup*  Supplementary

No significant between-group difference in brain area activation was found when viewing happy or sad faces vs. neutral faces.

## Discussion

The current study evaluated the translational implications of three different hV_1B_ variants by testing their activity in HEK293 transfected cells and their functional phenotype in a functional imaging task.

The experiments performed on transfected-cells indicated that PLC coupling was reduced in cells that expressed hV_1B_ K65N and/or R364H compared with WT V_1B_R. Fluorescence imaging performed using a selective hV_1B_ ligand d[Leu^4^-(Lys-Al647)^8^]VP indicate that the level of transfected hV_1B_ to HEK293 cells is statistically similar whatever the variant tested (see Fig. 1). Altogether these data signify that the differences in hV_1B_ coupling to PLC pathway observed for the different variants could not be the consequence of variable hV_1B_ R transfection since (i) our results are robust and very reproducible from one experiments to another (ii) the rate of transfection as well as the amount of fluorescence associated to plasma membrane are statistically similar whatever the variant tested. To comfort theses controls, we performed additional experiments by measuring the level of diacylglycerol (DAG) also produced by AVP by acting on hV_1B_. We found the same results, i.e. a reduction of DAG production in cells expressing hV_1B_ K65N and/or R364H, and and increase in cells expressing G191R [30].

These experiments thus indicated that the coupling between the hV_1B_ and the PLC is depending upon the hV_1B_ variant tested. As a consequence, if IP_3_ production is affected, DAG and intracellular calcium mobilization are also affected. Such modifications of all second messenger production may explain the functional physiological consequences observed for the different hV_1B_ variant studied.

The physiological role of AVP acting via central AVP1AR or AVP1BR receptors in regulating mood, memory, social, sexual and emotional behaviors is now well accepted [31]. Hernando et al. (2001) [32] first described the tissular localization of this receptor subtype within the rat brain using selective V_1B_R antibodies. We further confirmed and extended these data on the same biological model using the selective fluorescent V_1B_ agonist d[Leu^4^-(Lys-Al647)^8^]VP used in this study. V_1B_R are widely distributed in various regions of rat and mice brain (Olfactory bulb, lateral septum, Hipocampus CA2, Pyrifom cortex, Hypothalamus, Enthorinal cortex, Amygdala, Cingulate cortex…). Moreover, we described a direct coupling of rat V_1B_R to PLC on primary culture of rat pituitary cells [33]. The development of selective pharmacological tools able to discriminate the different AVP receptor subtype in each species have also allowed to demonstrate the essential role of V_1B_R in many physiological function [34]. For example, selective V_1B_ agonists and antagonists have also allowed the initial characterization of the V_1B_R subtype [6] and its role together with CRF in regulating the HPA axis by its effect on ACTH secretion at the pituitary level [6]. We also demonstrate by an electrophysiological single cell approach that in mousse CA2 region of the enthorinal cortex, AVP is able to modulate Long Term Potentiation [35]. More recently, in living mice, Leroy and collaborators elegantly demonstrated the role of V_1B_R located in the same brain region involved in regulating social aggression [36]. Both the development of new selective V_1B_R analogues as well the localization and pharmacological characterization of V_1B_R mutants found in different patient cohorts are thus of great importance for further translational studies.

In healthy participants, the fMRI facial emotion paradigm did not highlight any behavioral difference (reaction times for emotional faces and neutral faces); however, differences in brain activation were observed in participants harboring the hV_1B_ rs35369693 (K65N) and rs28632197 (R364H) polymorphisms. Indeed, upon stimulation with angry vs. neutral faces, activity in motor areas (supplementary motor cortex, putamen), ventral visual pathway areas (middle temporal gyrus, inferior temporal lobe) and hippocampus, precuneus and frontal sub-gyral area was increased in people with the GG genotype for hV_1B_ rs35369693 (K65N) compared with heterozygotes (CG genotype). Similarly, activity in the lingual gyrus and supplementary motor cortex was higher in participants with the GG than with the AG genotype for hV_1B_ rs28632197 (R364H).

Moreover, people with the GG genotype for these polymorphisms showed greater activation of motor and visual areas involved in face perception and recognition [37], when viewing angry faces. Although not significant, the reaction time also was longer in these participants during the angry face task. It could be hypothesized that overactivation of these areas may be an overcompensation system to respond as fast as possible during a cognitive task (sex recognition) when emotional information interferes.

In participants with the GG genotype for the hV_1B_ rs35369693 polymorphism (K65N), activation of hippocampus, putamen and frontal sub-gyral area was increased during the negative emotion recognition task. Interestingly, in rats, AVP is expressed in hippocampus, frontal and occipital lobe, and putamen [38]. Immunohistochemical and immunofluorescence analyses confirmed that V_1B_R receptors also are mainly localized in these regions [32, 39, 40]. Frontal sub-gyral functioning is impaired in schizophrenia during working memory tasks [41] and is implicated in heroin and internet addiction [42, 43]. Hippocampus and putamen are essential in emotion recognition [44, 45] through their involvement in the hate circuit [46] and angry face recognition [47]. Emotion recognition deficits are implicated in all mental disorders because of the role played by putamen and hippocampus in these disorders [48]. Notably, interactions between AVP and dopamine are involved in some of the social reward-related learning structures [49]. Indeed, previous research in mice showed that antagonizing V_1B_R mediate the protective effect of housing in the acquisition of morphine conditioned place-preference [50]. Thus, in individuals with the GG genotype for the hV1B rs35369693 polymorphism (K65N), V_1B_R may be implicated in functional working memory and/or reward-learning vulnerabilities. Moreover, individuals with the GG genotype for the hV_1B_ rs35369693 polymorphism (K65N) may be more sensitive to emotional information, especially anger. Both hV_1B_ rs35369693 (K65N) and rs33990840 (R364H) SNPs have been associated with autism spectrum disorder characterized by impaired emotion recognition [51] and with autistic traits in a non-clinical sample [52].

Due to the lack of heterozygotes for the hV_1B_ rs28632197 (G191R) SNP, fMRI analyses could not be performed in participants harbouring this polymorphism. In a study with 459 patients with depression, only 78 presented the AA/AG genotype for rs28632197, indicating that it is not common [53]. Importantly, a previous study reported that this genotype is more frequent in suicide attempters [14]. In our study, cells expressing hV_1B_ G191R showed the greatest IP activity. hV_1B_ is crucial in HPA axis regulation. In rats, ACTH and cortisol levels after some types of acute stress and after chronic stress are lower in animals in which V_1B_R was knocked out than in wild type controls [54–56], suggesting an anxiolytic effect of AVP suppression [55]. Thus, the very high IPs activity observed in cells expressing hV_1B_ G191R may suggest that this variant is implicated in the dysregulated HPA axis response described in patients who committed or attempted suicide [57–59]. Thus, hV_1B_ may be a pharmacological target for people with suicidal behaviors. V_1B_R antagonists, such as SSR149415, have already been tested in a mouse model of excessive alcohol drinking [60], and may help to regulate the HPA axis in patients harboring the hV_1B_ rs28632197 SNP and at suicidal risk.

This study has several limitations. The small sample for the fMRI study increased the risk of type-I and type-II error and limited the application of stringent corrections [61]. As the number of participants with the AA/AG genotype for the hV_1B_ rs28632197 SNP was too small, differences in emotional recognition could not be investigated for this SNP, a crucial issue in suicide, mood disorders and anxiety disorders. Moreover, the inclusion of only men in the fMRI study prevented the generalization of the obtained results.

Nonetheless, this study brought some information on the molecular and brain functioning of different hV_1B_ genotypes that are relevant to psychiatric conditions. Specifically, this study showed that IPs activation was reduced in cells expressing hV_1B_ K65N and R364H and that functional activation of areas related with emotional recognition was higher in participants with the GG genotype for hV_1B_ rs35369693 (K65N) and for hV_1B_ rs28632197 (R364H). Conversely, the highest IPs accumulation was observed in cells expressing hV_1B_ G191R, and this finding could be related to HPA axis dysregulation.

## Data Availability

The data that support the findings of this study are available on request from the corresponding author.
